# Characterization of eDNA from the Clinical Strain *Acinetobacter baumannii* AIIMS 7 and Its Role in Biofilm Formation

**DOI:** 10.1100/2012/973436

**Published:** 2012-04-19

**Authors:** Praveen K. Sahu, Pavithra S. Iyer, Amrita M. Oak, Karishma R. Pardesi, Balu A. Chopade

**Affiliations:** ^1^Institute of Bioinformatics and Biotechnology, University of Pune, Pune 411007, India; ^2^Department of Microbiology, University of Pune, Pune 411007, India

## Abstract

Release of extracellular DNA (eDNA) was observed during *in vitro* growth of a clinical strain of *Acinetobacter baumannii*. Membrane vesicles (MV) of varying diameter (20–200 nm) containing DNA were found to be released by transmission electron microscopy (TEM) and atomic force microscopy (AFM). An assessment of the characteristics of the eDNA with respect to size, digestion pattern by DNase I/restriction enzymes, and PCR-sequencing, indicates a high similarity with genomic DNA. Role of eDNA in static biofilm formed on polystyrene surface was evaluated by biofilm augmentation assay using eDNA available in different preparations, for example, whole cell lysate, cell-free supernatant, MV suspension, and purified eDNA. Biofilm augmentation was seen up to 224.64%, whereas biofilm inhibition was 59.41% after DNase I treatment: confirming that eDNA facilitates biofilm formation in *A. baumannii*. This is the first paper elucidating the characteristics and role of eDNA in *A. baumannii* biofilm, which may provide new insights into its pathogenesis.

## 1. Introduction

Pathogenesis and multidrug resistance of *Acinetobacter baumannii* has been a serious concern in the management of infections caused by the organism worldwide. It contributes to 2–10% of all Gram negative infections and 9% of total nosocomial infections [1, 2]. Associated mortality up to 30% [3] is seen with *A. baumannii *infections such as ventilator-associated pneumonia, bacteraemia, urinary tract infections, burn wound infections, endocarditis, secondary meningitis, and septicemia, especially in intensive care units [2, 4, 5]. *A. baumannii *has the capacity of acquiring putative genetic factors as plasmids and pathogenicity islands and exhibits high-level of multidrug, and metal resistance [6, 7]. Global rise of multidrug-resistant *A. baumannii *[8], therefore, poses a major challenge to current treatment options. Biofilm formation is considered as a factor contributing to the pathogenicity of *A. baumannii,* and it imparts high levels of drug resistance that lead to treatment failure. The capacity of this bacterium to adhere to epithelial cells is due to a positive correlation of biofilm formation with adherence [9] and probably explains the clinical success of *A. baumannii *[10]. In *A. baumannii* ATCC 19606, a two-component regulatory system *bfmRS* is found to play an important role in biofilm formation and cellular morphology [11]. 

Bacterial cell aggregation and biofilm formation on surfaces is a complex process that involves a series of highly regulated molecular events and the participation of multiple determinants. These structures are found encased in an extracellular matrix composed of carbohydrates and polysaccharides, proteins, other macromolecules, and nucleic acids, for example, DNA and RNA [12]. It has been seen that a significant fraction of the biofilm matrix can be only DNA. For instance, extracellular DNA can be up to 50% more abundant than cellular DNA in unsaturated biofilms of *Pseudomonas aeruginosa *[13]. eDNA was first demonstrated to be a matrix component of *P. aeruginosa* biofilms [14]. It was also reported that eDNA originates from the intracellular DNA under conditions in which lysis is not observed [15]. In case of *Staphylococcus epidermidis *[16] and *Enterococcus faecalis *[17], eDNA results from the autolysin-mediated killing of a small subpopulation of cells, to provide DNA as a component of biofilm matrix. A recent study shows that eDNA can be released via autolysis and can be a major contributor to vancomycin-enhanced biofilm formation in *Staphylococcus aureus *[18].

Biofilm formation by *A. baumannii *[19, 20] has facilitated its survival on dry surface and enhanced antibiotic resistance, resulting in *A. baumannii* as a persistent infectious agent in intensive care units (ICUs). Our recent investigation [21] shows that biofilm formation by *A. baumannii* strains on clinical devices, such as urinary catheters, could explain their ability to persist in clinical environments and their role in device-related infections. Nevertheless, the available knowledge on the specific molecular determinants in the development of biofilms in *A. baumannii* is scarce. We hypothesize that eDNA could be one such critical determinant. Although studies on eDNA and its role in natural transformation in *Acinetobacter* have been reported earlier [22–25], to date eDNA remains an uncharacterized determinant in the pathogenic bacterium *A. baumannii*. Since biofilm formation is a major virulence factor in *A. baumannii*, characterization of eDNA from *A. baumannii* with regard to biofilm development is worthwhile investigating. In this study, we have characterized eDNA from a multidrug-resistant clinical strain of *A. baumannii* and demonstrated its role in *in vitro* biofilm formation on abiotic surfaces. 

## 2. Material and Methods

### 2.1. Bacterial Culture and Growth Conditions

A clinical strain of* Acinetobacter baumannii *AIIMS 7, previously isolated [26], was used in this study. The species level identification was done by chromosomal DNA transformation assay, API 32 GN System [27, 28] and confirmed by *16SrRNA* gene sequencing (GenBank accession EU779829). Phenotypic identification was performed using biochemical assays [29]. The bacterium was grown and maintained on cysteine-lactose electrolyte deficient (C.L.E.D.) agar and in Luria broth (HiMedia, India) with appropriate antibiotics at 37°C. The antibiotic resistance profile was determined according to Clinical and Laboratory Standards Institute (CLSI) protocols. Growth curve of *A. baumannii* AIIMS 7 was analyzed up to 96 hours, by taking absorbance at 600 nm in a UV-Vis spectrophotometer (Shimadzu, Japan).

### 2.2. Purification of eDNA in Temporal Scale of Growth

eDNA was purified from cell-free supernatant filtered through 0.22 *μ*m syringe filters (PALL, USA) sampled at various time points of growth up to 96 hours using methods described elsewhere [30] with modifications. Briefly, 750 *μ*L of cell-free supernatant was added to an equal volume of buffer-A (50 mM Tris, 10 mM EDTA with 1% cetyl trimethyl ammonium bromide (CTAB), pH 8.0 and incubated at 65°C for 30 min, followed by centrifugation at 6500 g for 10 min. To the pellet, 500 *μ*L of buffer-B (10 mM Tris, 0.1 mM EDTA and 1 M NaCl, pH 8.0.) was added followed by 0.3 volumes of ice-cold isopropanol. After incubation for three hours at 4°C, the precipitated DNA was centrifuged at 12000 g for 15 min, to obtain the pellet which was finally resuspended in 40 *μ*L of DNase RNase free Tris-EDTA buffer (10 mM Tris-Cl pH 8.0, 1 mM EDTA, Sigma Aldrich, USA). The pellet was solubilised at 4°C overnight. To eliminate proteins, samples were treated with Proteinase K (10 mg/mL, Sigma Aldrich, USA) and incubated at 37°C for one hour and reprecipitated with ice-cold isopropanol. Genomic DNA was also purified using a commercial kit (Sigma Aldrich, USA). Concentrations of DNA were determined in a Biophotometer Plus (Eppendorf, Germany).

### 2.3. Purification of Membrane Vesicles

To check whether total eDNA found in cell-free supernatant originates from membrane vesicles, their presence was tested during active growth phase of *A. baumannii* AIIMS 7. Membrane vesicle purification was done from cell-free supernatant as per methods described elsewhere [31]. Briefly, Luria broth (150 mL) was inoculated with 10^6 ^CFU/mL of *A. baumannii *AIIMS 7 and incubated at 37°C for 15 hours at 150 rpm. Cells were pelleted and supernatant was sequentially filtered through a 0.45 *μ*m and a 0.22 *μ*m pore filter (PALL, USA). Filtered cell-free supernatant was subjected to ultracentrifugation (141,000 g, three hours, 4°C) in an Ultracentrifuge (Beckmann, USA). Pellet was resuspended in 50 mM of 4-(2-hydroxyethyl)-1-piperazineethanesulfonic acid (HEPES) buffer supplemented with 0.5 mM dithiothreitol (DTT) (HiMedia, India). The purified membrane vesicle suspension was treated with DNase I (10 mg/mL, Sigma Aldrich) to remove any unencapsulated eDNA, was again filtered through a 0.22 *μ*m syringe filter to remove any cells leftover, and was stored at −20°C until use.

### 2.4. Visualization of Membrane Vesicles by Electron Microscopy

The purified membrane vesicle suspension was localized by transmission electron microscopy using methods described earlier [32]. Briefly, 20 *μ*L purified membrane vesicle suspension was placed on carbon and Formvar precoated copper grids, washed by floating in 50 *μ*L of molecular grade water and stained with 2% uranyl acetate and dried. Stained grids were examined in a Transmission Electron Microscope (JEOL, Japan) operating under standard conditions. For atomic force microscopy, 10 *μ*L of purified membrane vesicle suspension was placed on freshly cleaved mica for 1 min and air dried. To minimize damage to membrane vesicles, a small deflection signal of −0.5 V (operating in contact mode) was applied and visualized with a slow scan rate of 0.25 to 0.50 Hz in an Atomic Force Microscope (JEOL, Japan).

### 2.5. Digestion of eDNA by DNase I and Restriction Enzymes

To check the digestion pattern of eDNA and compare with genomic DNA, eDNA was digested with six different restriction enzymes *Bam*HI, *Eco*RI, *Hin*dIII, *Pst*I, *Xba*I, and *Xho*I (Sigma Aldrich). Briefly, 5 *μ*g of eDNA were mixed with five units of each restriction enzymes along with respective enzyme reaction buffers and bovine serum albumin (100 mg/mL) and incubated at 37°C for two hours. eDNA was also treated with DNase I (10 mg/mL, Sigma Aldrich), RNase A (10 mg/mL, Sigma Aldrich, USA), and Proteinase K (10 mg/mL, Sigma Aldrich, USA). The digested DNA fragments were separated by agarose gel electrophoresis and documented in a Gel Documentation system (Alpha Innotech, USA). Results were compared with those of genomic DNA.

### 2.6. Amplification of 16SrRNA, 5′coding Region of bap and *bla*_*PER*-1_

*In vitro* amplification was performed for detection of *16SrRNA* and *bla*_*PER*-1_ (encoding PER-1 type extended spectrum *β*-lactamases) in eDNA using PCR primers 16S-F/R and bla-F/R [9], and a 5′coding region of biofilm-associated protein (1019 bp, position: 152-1171-c) using primers 1124/1125 [33]. Annealing temperatures were used for PCR according to each primer set, that is*, 16SrRNA*: 63°C, *bap*: 60.5°C, and *bla*_*PER*-1_: 36.5°C. The final PCR program used was 94°C for 5 min, 40 cycles at 94°C for 45 sec, 63°C/60.5°C/36.5°C for 45 sec, 72°C for 60 sec/60 sec/90 sec, and final extension at 72°C for 5 min, respectively, for *16SrRNA*, *bap,* and *bla*_*PER*-1_. PCR reagents and primers were sourced from Sigma Aldrich, USA, and assayed in a gradient PCR (Eppendorf, Germany). All PCR assays were repeated at least thrice, using DNase, RNase, and protease free water (Bangalore GeNei, India) as a negative control and genomic DNA as a positive PCR control. PCR fragments were electrophoresed in 1.5% agarose gels stained with 0.5 *μ*g/mL ethidium bromide and photographed in Gel Documentation system (Alpha Innotech, USA). Amplicons were purified using a gel extraction kit (Sigma Aldrich, USA) according to manufacturer's instructions.

### 2.7. DNA Sequencing

Purified PCR products were sequenced in a DNA Analyzer using Big Dye Terminator cycle sequencing reaction kit (Applied Biosystems, USA). Sequencing products were purified using a commercial kit (Applied Biosystems, USA) as per manufacturer's instructions. Sequences were analyzed using Sequencing Analysis Software (Applied Biosystems, USA). All Sequencing reactions were performed in triplicates. *16SrRNA* and 5′coding region of *bap* sequences were submitted to GenBank, NCBI (National Centre for Biotechnology Information, USA).

### 2.8. Screening for Presence of Integrative Phages

To check for the presence of integrative phages which may contribute to the total available eDNA in the cell-free supernatant, pro-phage induction in *A. baumannii* AIIMS 7 was investigated by UV irradiation and Mitomycin C, according to methods described [34] with required modifications. Briefly, an overnight grown culture of *A. baumannii* AIIMS 7 was diluted 1 : 100 in Luria broth and absorbance was taken at 600 nm, followed by incubation in a water bath for 30 min at 37°C. Mitomycin C was added to a final concentration of 0.5 *μ*g/mL and incubated for six hours monitoring the absorbance at each hour. The suspension was centrifuged at 3000 g for 12 min and the supernatant was collected. To investigate UV irradiation-induced prophages, an overnight grown culture of *A. baumannii* AIIMS 7 was diluted 1 : 50 in Luria broth and incubated at 37°C for three hours, followed by centrifugation at 6000 g for 10 min. The pellet was resuspended in 100 mM MgSO_4_ and transferred to a sterile glass Petri plate followed by irradiation for 30 seconds by keeping at 16 cm from the germicidal short wave lamp. The supernatants from both experiments were tested for prophage presence by spot test and double-layered plaque assay.

### 2.9. Test for Natural Transformation

It was necessary to evaluate whether *A. baumannii *AIIMS 7 undergoes natural transformation, since eDNA could be associated with the process. To rule out the probability, natural transformation was tested in *A. baumannii *AIIMS 7 according to methods described earlier [35].

### 2.10. Biofilm Augmentation Assay

To demonstrate the role of eDNA in biofilm development, overall increase in biofilm matrix was taken as a measure for calculating biofilm augmentation. Five different preparations containing eDNA were chosen on the basis that they contain eDNA in one or more forms. The preparations were, namely, purified eDNA, cell-free supernatant (CFS), concentrated CFS (15× CFS), whole cell lysate, and membrane vesicle suspension. Purified eDNA and membrane vesicle suspension were prepared according to methods described earlier and were used for the assays. Cell-free supernatant (CFS) was directly used in biofilm augmentation. It was further concentrated to 15 times (15×), by freeze drying for 14 hours in a lyophilizer (Thermo, USA). Whole cell lysate was prepared taking the cell pellet (10 mg dry weight) from overnight grown suspension culture. The pellet was resuspended with 200 *μ*L molecular grade water and heated for 20 min at 100°C. The lysate was then sonicated and vortexed rigorously for 10 min. Presence of eDNA was checked in all the preparations, by agarose gel electrophoresis, and results were documented in Gel Documentation system (Alpha Innotech, USA). Membrane vesicles were lysed by heat treatment and treating with 1% sodium dodecyl sulphate (SDS) prior to loading on the agarose gel. Luria broth was loaded as negative control.

Biofilm quantification was done according to methods described elsewhere [9]. Overnight grown culture of *A. baumannii* AIIMS 7 (10^6 ^CFU/mL) was inoculated onto single microtitre wells with final dilution of 1 : 40 with sterile Luria broth and incubated at 37°C for biofilm development as control sample. Cell-free supernatant was replaced with Luria broth for the first variation, whereas 20 *μ*L each of whole cell lysate, 15× CFS, membrane vesicle suspension and purified eDNA were individually supplemented to Luria broth and inoculated with 10^6 ^CFU/mL cells. Negative controls (no cells) were included for each of the experimental preparations. The microtitre plates were then incubated overnight at 37°C under static conditions and processed thereafter. Nonadherent cells were removed from microtitre wells by sonication followed by aspiration. The wells containing biofilm matrices were washed thrice with sterile PBS and stained with 0.1% gentian violet (HiMedia, India) for 10 min at room temperature. Excess stain was removed by immersing in a water trough and dried in laminar air flow. Finally, 200 *μ*L of absolute ethanol was added to each well shaken at 1020 rpm for 10 seconds. Absorbance at 570 nm was recorded in a Multi-Plate Reader (Molecular Devices, USA). Biofilm indices were calculated after normalizing with appropriate controls. All biofilm assays were repeated thrice (in replicates of twelve for each variation).

### 2.11. Treatment of Preformed Biofilms with DNase I

To investigate the role of eDNA during biofilm development, preformed biofilms of *A. baumannii* AIIMS 7 on polystyrene microtitre wells were treated with 2 mg/mL DNase I (Sigma Aldrich, USA). Biofilm formation was quantified according to methods described previously [9].

### 2.12. Statistical Analysis

Results obtained (in replicates) from nucleic acid quantification and biofilm assays were entered in to excel spreadsheets (Microsoft, USA). Frequency distributions, namely, mean with standard deviations were determined. Statistical analysis was performed by Student's two tailed *t*-test, and *P* value < 0.05 was considered to be statistically significant.

### 2.13. Nucleotide Sequence Accession Numbers

The accession numbers of *16SrRNA* and 5′coding region of *bap* sequences (amplified from eDNA) was HM992508 and HM765514, respectively.

## 3. Results

### 3.1. Presence of eDNA in Extracellular Growth Medium

**Figure 1:**
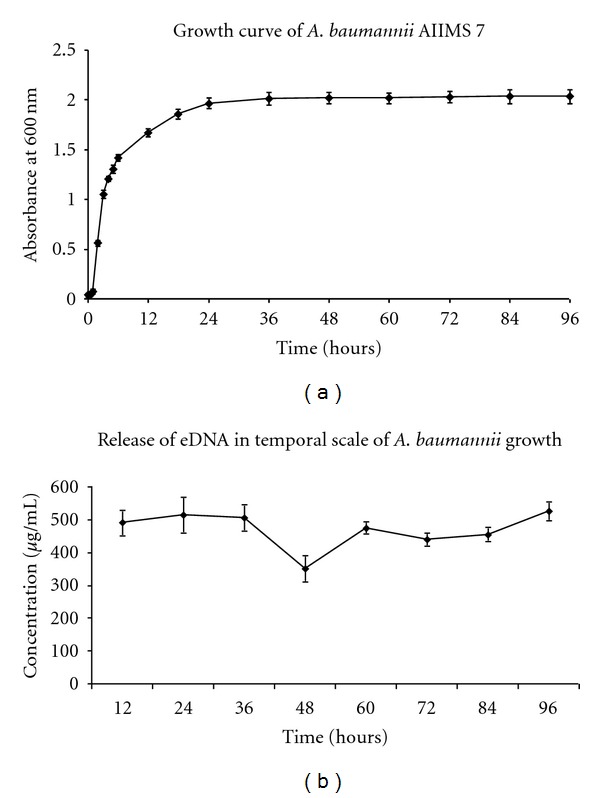
(a) Growth curve of *Acinetobacter baumannii* AIIMS 7. (b) Pattern of eDNA release in temporal scale of *A. baumannii* AIIMS 7 growth. Graph representing concentrations of eDNA purified from cell-free supernatant of *A. baumannii* AIIMS 7 at respective time points. The pattern shows a depression at 48 hour, otherwise showing almost steady presence of eDNA in extracellular medium. (Concentrations of eDNA: 490 ± 54.74, 515 ± 40, 505 ± 39.05, 350 ± 17.55, 475 ± 20.56, 440 ± 20.61, 455 ± 27.83, 525 ± 44.43 *μ*g/mL, respectively, at 12, 24, 36, 48, 60, 72, 84, and 96 hours).

To check the presence of DNA in the extracellular growth medium of *A. baumannii* AIIMS 7, eDNA was purified by isopropanol precipitation at ice-cold temperature. eDNA was found to be present along the temporal scale of *A. baumannii* AIIMS 7 growth up to 96 hours and showed a pattern as shown in graph (Figure 1(b)). The pattern of eDNA (Figure 1(b)) showed almost similar concentrations of eDNA in the early growth phase (up to 36 hours), a fall in the mid-stationary phase (48 hour), followed by steady concentrations in the late growth phases (60 to 96 hours). Correlation of growth curve (Figure 1(a)) and concentration of purified eDNA showed that the prevalence of eDNA was almost consistent. Concentrations of eDNA were comparable to earlier findings in *Pseudomonas aeruginosa *[15]. 

### 3.2. Membrane Vesicles Present in Extracellular Growth Medium Contained DNA

**Figure 2:**
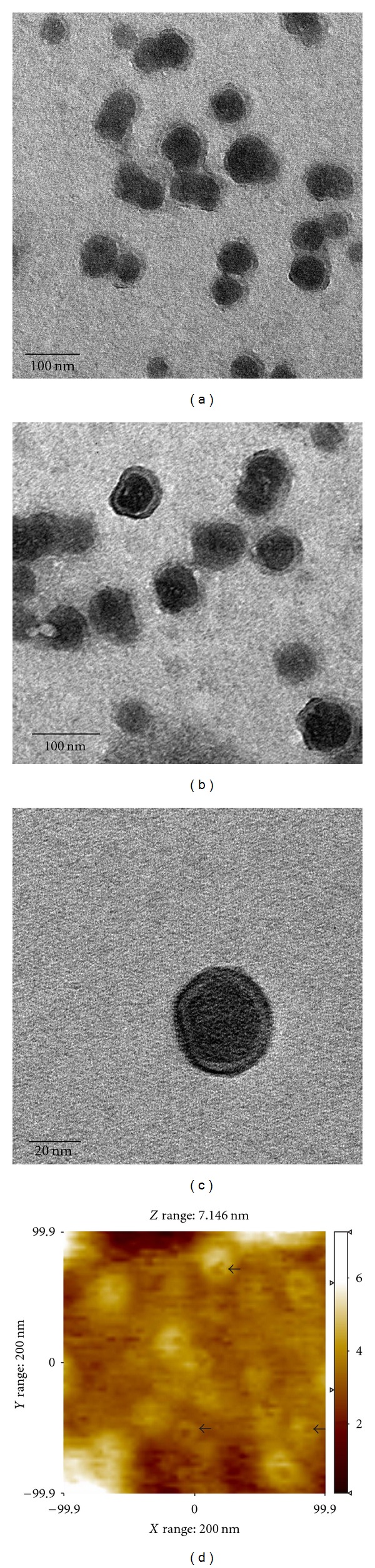
Visualization of membrane vesicles released from *A. baumannii *AIIMS 7 by Transmission Electron and Atomic Force Microscopy. (a-b): Transmission Electron Micrographs of membrane vesicles purified from *Acinetobacter baumannii *AIIMS 7, after negative staining with Uranyl acetate. (Bar, 100 nm). (c): Single membrane vesicle showing clear outer vesicular membrane with electron-dense core, indicating presence of nucleic acid. (Bar, 20 nm). (d): Atomic Force Micrograph of membrane vesicle suspension (*X*, *Y* range: 200 nm, *Z* range: 7.146 nm). Arrows indicate individual membrane vesicles.

Membrane vesicles were purified from cell-free supernatant (early growth phase) and characterized using electron microscopy to determine their size and morphology. Electron microscopy revealed presence of membrane vesicles of varying diameter from 20 to 200 nm (Figures 2(a)–2(c)) and atomic force microscopy confirmed the dimensions as seen in the two-dimensional picture (Figure 2(d)). 

They were seen as round, regular structures, with bi-layered membrane encapsulating electron-dense material (Figure 2(c)). Uranyl acetate was used for negative staining of membrane vesicles, which preferentially stains nucleic acids. The dark stained portion in the core could be indicative of membrane vesicles containing nucleic acids, which could be DNA, RNA, and even plasmids. In the literature, membrane vesicles are reported to contain nucleic acid and even functional enzymes. It was further confirmed when DNA was visualized in an agarose gel when lysed membrane vesicle suspension was loaded (Figure 5, Lane 3). The size of DNA found inside membrane vesicles was similar to that of eDNA (Figure 5, Lane 2) and, therefore, could be a result of encapsulation during the process of vesiculation.

### 3.3. Absence of Integrative Phages Indicated eDNA to Be Exclusively of Bacterial Origin

Both spot test and double-layered plaque assay showed negative results: no plaques were observed even 96 hours after incubation at 37°C confirming absence of integrative phages in *A. baumannii *AIIMS 7. This indicates that eDNA characterized from cell-free supernatant was exclusively from this organism. The exclusivity of the origin of eDNA in the medium was further confirmed by amplifying the *A. baumannii 16SrRNA *gene by PCR.

### 3.4. *A. baumannii* AIIMS 7 Did Not Exhibit Natural Transformation

*A. baumannii *AIIMS 7 did not show ability to undergo natural transformation unlike several members of the genus *Acinetobacter* capable of uptake and release of DNA [22]. In naturally competent strains, extracellular DNA could be found in the extracellular environment, for the accomplishment of natural transformation events, for example, induction of competence, translocation of DNA with the competence protein machinery, and finally transport across the membrane for both uptake as well as release. But *A. baumannii* strains have rarely been seen to undergo natural transformation and similar results were obtained with *A. baumannii *AIIMS 7. This suggested that the eDNA was released via processes other than natural transformation.

### 3.5. Characteristics of eDNA Were Similar to Genomic DNA

**Figure 3:**
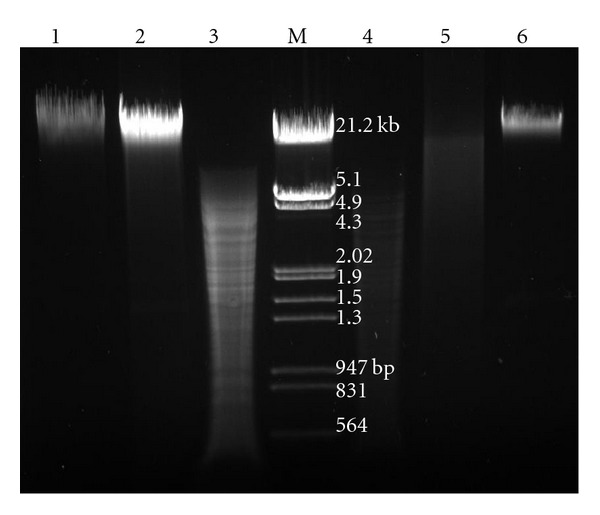
Comparison of digestion pattern of eDNA and genomic DNA (by DNase I and restriction enzymes). Agarose gel (0.8%) showing Lane 1: Genomic DNA, Lane 2: Digestion of genomic DNA with DNase I, Lane 3: Digestion of genomic DNA with restriction enzymes (*Bam*HI, *Eco*RI, *Hin*dIII, *Pst*I, *Xba*I, *Xho*I), Lane M: Molecular weight marker (*λ* phage genome *Eco*RI*/Hin*dIII digest), Lane 4: Digestion of eDNA with restriction enzymes (*Bam*HI, *Eco*RI, *Hin*dIII, *Pst*I, *Xba*I, *Xho*I), Lane 5: Digestion of eDNA with DNase I, Lane 6: Purified eDNA.

**Figure 4:**
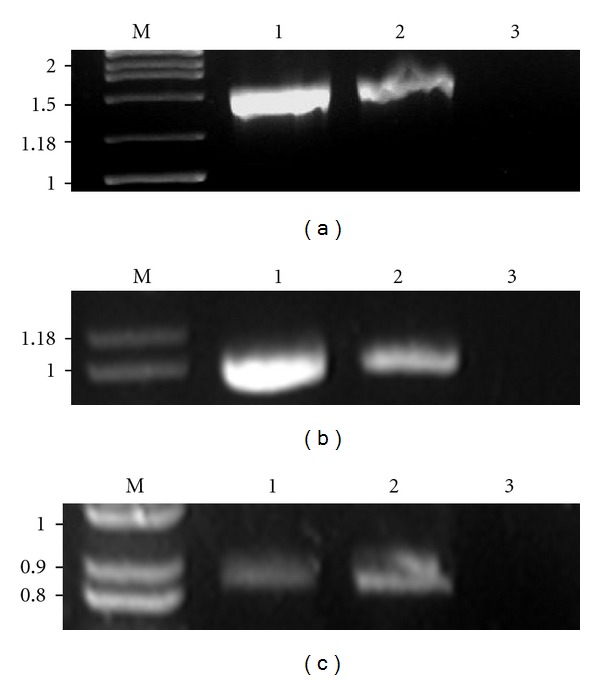
PCR amplification of *16SrRNA,* 5′coding region of *bap* and *bla*_*PER*-1_ from genomic DNA and eDNA. Agarose gel (1.5%) showing (a): Lane M: Molecular weight marker (values in kb), Lane 1: *16SrRNA* (1.5 kb) amplified from genomic DNA, and Lane 2: *16SrRNA* (1.5 kb) amplified from eDNA. Lane 3: DNase, RNase, Protease-free water as negative PCR control. (b): Lane M: Molecular weight marker (values in kb), Lane 1: 1019 bp sized 5′coding region of *bap* gene amplified from genomic DNA, and Lane 2: 1019 bp sized 5′coding region of *bap* gene amplified from eDNA, Lane 3: DNase, RNase, Protease-free water as negative PCR control. (c): Lane M: Molecular weight marker (values in kb), Lane 1: 875 bp *bla*_*PER*-1_ gene amplified from genomic DNA, Lane 2: 875 bp *bla*_*PER*-1_ gene amplified from eDNA, and Lane 3: DNase, RNase, Protease-free water as negative PCR control.

**Figure 5:**
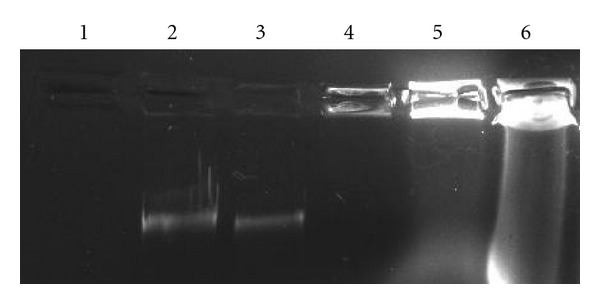
Analysis of eDNA present in various preparations used for biofilm augmentation. Agarose gel (0.8%) showing Lane 1: Luria broth control, Lane 2: purified eDNA, Lane 3: lysed membrane vesicle suspension, Lane 4: cell-free supernatant (CFS), Lane 5: concentrated cell-free supernatant (15× CFS), and Lane 6: whole cell lysate. DNA can be seen well separated on agarose gel (Lanes 2,3: purified eDNA and DNA from membrane vesicles); however, in case of CFS, 15× CFS, and whole cell lysate (lane 4, 5, 6.), it can be seen in the wells.

The digestion pattern of eDNA with DNase I and restriction enzymes (*Bam*HI, *Eco*RI, *Hin*dIII, *Pst*I, *Xba*I, *Xho*I) was similar to genomic DNA. Agarose gel electrophoresis indicated that the size of eDNA is comparable to that of genomic DNA (>21.2 kb). eDNA was completely digested with DNaseI (Figure 3), but remained unaffected under RNase and Proteinase K. PCR amplification of *16SrRNA*, *bla*_*PER*-1_, and *bap* from eDNA showed bands and sizes (1.5 kb, 837 bp, and 1025 bp, resp.) on agarose gel similar to those from the genomic DNA of *A. baumannii* AIIMS 7 (Figures 4(a)–4(c)).

### 3.6. *A. baumannii* Biofilms Were Significantly Augmented by eDNA

**Figure 6:**
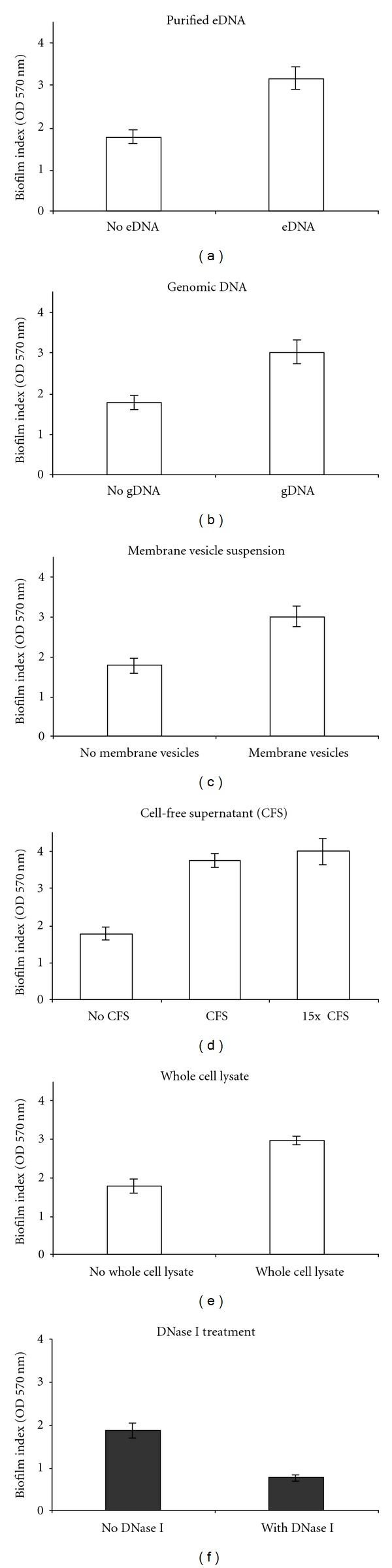
Quantitative biofilm assays for *A. baumannii *AIIMS 7. Representative graphs showing biofilm augmentation after external supplementation with (a): purified eDNA (178.83% augmentation); (b): purified genomic DNA (170.79% augmentation); (c): purified membrane vesicle suspension (170.11% augmentation); (e): whole cell lysate (167.89% augmentation); (d): biofilm augmentation seen after replacing Luria broth with cell-free supernatant (CFS; 211.19% augmentation) and supplementation with 15× CFS (224.64% augmentation) (f): Effect of DNase I treatment on preformed biofilms. (59.41% reduction).

We were interested in studying the effect on biofilm development when DNA was made available externally. We supplemented biofilms with eDNA in various preparations to mimic its natural availability during growth. Figures 6(a)–6(e) shows that eDNA supplemented in any of the given forms was able to augment the biofilms on polystyrene microtitre surface significantly. However, there was a mild variation in biofilm augmentation capabilities of the supplements which contained eDNA. Similar levels of augmentation were observed with membrane vesicles containing DNA, whole cell lysate, and purified eDNA and genomic DNA (Figures 6(a), 6(b), 6(c), and 6(e)). Cell-free supernatant (CFS) and its concentrated form (15× CFS) conferred maximum biofilm augmentation (211.19% and 224.64%, resp., Figure 6(d)). Overall, based on the results of biofilm augmentation, it was inferred that presence of eDNA in the vicinity of cells attached to surface can favorably lead to a more abundant development of biofilm, which may vary upon the environment and conditions in which eDNA is present.

### 3.7. Preformed Biofilm of *A. baumannii* Was Inhibited by DNase I

Treatment of preformed *A. baumannii* biofilms with DNase I showed reduction in biofilm biomass up to 59.41% (Figure 6(f)). In several bacterial biofilms, presence of DNA in the biofilm matrix makes the biofilm stable due to its polymeric and polyionic nature. This experiment explained that deficiency of DNA in the biofilm matrix can lead to severe reduction of biofilm biomass. It is also direct evidence that abiotic (polystyrene) surface biofilms of *A. baumannii *AIIMS 7 were significantly dependent upon the presence of eDNA.

## 4. Discussion

Extracellular DNA can be of genomic origin [35], and its role has been reported earlier in several other bacteria, but there are no reports as yet in *A. baumannii*. In our study, eDNA has been characterized from a clinical strain of *A. baumannii* and its role in biofilm development is demonstrated for the first time. The characteristics of eDNA were very similar to those of genomic DNA, as seen in agarose gels, and further confirmed by *in vitro* amplification of *16SrRNA,* *bla*_*PER*-1_, and 5′coding region of *bap*. This is in agreement with studies [16] which showed amplification of several *Staphylococcus epidermidis* genes from its eDNA and compared with genomic DNA. Similarly, in *Pseudomonas aeruginosa* and *Pseudomonas putida* biofilms, eDNA is similar to genomic DNA [13]. eDNA can serve as raw material for gene transfer [23], as in the case of naturally transformable bacteria like *Acinetobacter* sp.BD413. However, this was not the case of *A. baumannii* AIIMS 7 since the strain did not exhibit natural transformation.

During *in vitro* growth of *A. baumannii*, we analyzed the concentrations of eDNA available in the extracellular medium (cell-free supernatant) up to 96 hours, which described a novel pattern of prevalence. Based on eDNA release pattern and growth curve in the temporal scale (Figures 1(a) and 1(b), the origin of eDNA in *A. baumannii* AIIMS 7 was attributed to three major processes: (i) active release of eDNA in free form, (ii) encapsulation of eDNA by membrane vesicles released (early growth stages), and (iii) passive release of eDNA during cell lysis (later stages of growth).

- *A. baumannii *eDNA at early stages indicates active release of DNA which is independent of lysis. Comparable findings are described in earlier studies in *P. aeruginosa *[15]. Autolysin-mediated eDNA release and biofilm formation is shown in *Enterococcus faecalis and Staphylococcus epidermidis *[16, 17]. It is interesting to speculate whether or not *A. baumannii *harbors a mechanism for eDNA release by regulated lysis of a subpopulation to promote biofilm formation. No experimental data records exist to our knowledge, except presence of an autolysin-like ORF in whole genome map of *A. baumannii* AB0057 [36]. A fall in eDNA concentration seen at mid-stationary phase (48 hours), however, remained unclear. Nutrient limitation, growth inhibition, accumulation of secondary metabolites, and probable downregulation of putative autolysin genes could be probable reasons.
- Membrane vesicles are released during *in vitro* growth of *A. baumannii, *according to recent studies [37, 38]. Here, we report release of membrane vesicles for the first time, seen in an Indian clinical isolate *A. baumannii* AIIMS 7. These MVs contained DNA, which was confirmed by localization by electron microscopy after uranyl acetate staining, and subsequently by electrophoresis of vesicular DNA. Vesicular DNA has been reported to be amplifiable in case of other Gram-negative bacteria [39] such as *Escherichia coli* O157:H7 [31] and *Pseudomonas aeruginosa* PA01 [40] and was seen in this study as well (*16SrRNA*, *bla*_*PER*-1_ genes; data not shown). The electron dense material present inside membrane vesicles explains that DNA could be encapsulated during active growth; therefore, release of membrane vesicles into the extracellular growth medium can contribute significantly to the total availability of eDNA.
- Any eDNA seen during the log phase of growth should mostly be due to active secretion mechanisms and not due to cell death. High concentration of eDNA at later stages of growth (post-48 hours) can be attributed to cell lysis leading to passive release of DNA into the extracellular medium. *16SrRNA* amplification from eDNA confirmed that the DNA available in the medium may comprise fractions of genomic DNA. Nevertheless, the actively released eDNA, membrane vesicles (containing DNA), and putative autolysin-mediated eDNA release would account for the major fraction of total eDNA at the earlier stages of growth (up to 36 hours).

eDNA is a matrix component of microbial biofilms [41] and is involved in the process of biofilm formation, as earlier demonstrated in several bacteria, for example, *Bacillus cereus, Staphylococcus epidermidis, *and* Enterococcus faecalis *[16, 17, 42]. eDNA is essential for initiation and stabilization of biofilms. As reviewed earlier [43], bacterial eDNA (present as a component of the biofilm EPS) is involved in essential functions such as initial adhesion, aggregation of cells, and cohesion of biofilms to provide mechanical stability. The role of eDNA in these developmental processes can be largely in combination with other components present in EPS such as polysaccharides, proteins, and amphiphilic molecules. In *Pseudomonas aeruginosa*, a similar phenomenon is observed where eDNA acts as a scaffolding agent [14] and an intracellular connector [44]. Being polyionic, eDNA can link other molecules together on the biofilm matrix as like teichoic acid. Similar observations have been reported in *Staphylococcus aureus* biofilms, which suggest that effect of DNase was observed due to the direct action of DNase on the biofilm matrix which contained DNA, independent of growth pattern or cell number [45]. Augmentation of biofilms by external supplementation of eDNA, and the inhibitory effect of DNase I on *A. baumannii* biofilms as shown here, proves that progressive biofilm development in *A. baumannii* is dependent on availability of eDNA.

Our study highlights the importance of eDNA demonstrated through biofilm augmentation assays. It demonstrates that irrespective of its origin whether from active eDNA release, contained inside membrane vesicles, or from natural cell lysis, eDNA is crucial for bacterial biofilms. eDNA can be efficiently taken up for building of biofilm matrix and can well serve as scaffolding agent during bacterial aggregation and stabilization of biofilms [14, 44]. eDNA also forms defined network-like structure during biofilm development, and, therefore, imparts stability [46]. We observed that the cell-free supernatant (in concentrated form, 15× CFS) shows maximum augmentation of biofilm, since eDNA present in free form can be easily made available across the surface of biofilm and has more chance of making scaffolds and thereby increasing the biomass by adhering to other matrix components by ionic interactions. The whole cell lysate supplementation mimicked the availability of eDNA at later stage, that is, passive release of DNA from lysed cells. It was seen to augment the biofilm (167.89%; Figure 6(e)) suggesting that DNA from culture in late growth phase may also contribute to growing or freshly dispersed biofilms. Membrane vesicles are known to help in biofilm enrichment, as shown in earlier studies in *P. aeruginosa *[40]. Similar results were seen with *A. baumannii *membrane vesicles (present study), which exhibited biofilm augmentation equivalent to that by purified genomic DNA, eDNA, or whole cell lysate.

Collectively, this work demonstrates that eDNA is present in the extracellular milieu, during *in vitro* growth of *A. baumannii *AIIMS 7. Besides originating from cell lysis at later stages, eDNA results from active release at early growth phases either in free form or contained in membrane vesicles of diameter 20–200 nm. eDNA in any of its natural forms is able to augment *A. baumannii* biofilms on an abiotic surface to significant levels, evident of having a role in progressive biofilm formation. Furthermore, preformed biofilms were inhibited by DNase I, supporting the role of eDNA in biofilms. DNA has been a target for inhibiting biofilms of *P. aeruginosa *[47] and, therefore, can have potential for combination therapy (with antibiotics) in the treatment of biofilm-associated infections caused by multidrug-resistant *A. baumannii*, which, however, warrants further experimental validation.
